# Ultrasonic Time-of-Flight Diffraction Imaging Enhancement for Pipeline Girth Weld Testing via Time-Domain Sparse Deconvolution and Frequency-Domain Synthetic Aperture Focusing

**DOI:** 10.3390/s25061932

**Published:** 2025-03-20

**Authors:** Eryong Wu, Ye Han, Bei Yu, Wei Zhou, Shaohua Tian

**Affiliations:** 1 Donghai Laboratory, Zhoushan 316021, China; wueryong@zju.edu.cn; 2 Ocean Research Center of Zhoushan, Zhejiang University, Zhoushan 316021, China; 3 PipeChina (Xuzhou) Pipeline Inspection and Testing Co., Ltd., Xuzhou 221008, China; hanye02@pipechina.com.cn; 4 School of Mechanical Engineering, Zhejiang University, Zhoushan 316021, China; yubei@zju.edu.cn (B.Y.); 22260749@zju.edu.cn (W.Z.); 5 Hangzhou Shenhao Technology Co., Ltd., Hangzhou 311113, China

**Keywords:** ultrasonic imaging, time-of-flight diffraction (TOFD), sparse deconvolution, synthetic aperture focusing technique (SAFT), image resolution enhancement

## Abstract

Ultrasonic TOFD imaging, as an important non-destructive testing method, has a wide range of applications in pipeline girth weld inspection and testing. Due to the limited bandwidth of ultrasonic transducers, near-surface defects in the weld are masked and cannot be recognized, resulting in poor longitudinal resolution. Affected by the inherent diffraction effect of scattered acoustic waves, defect images have noticeable trailing, resulting in poor transverse resolution of TOFD imaging and making quantitative defect detection difficult. In this paper, based on the assumption of the sparseness of ultrasonic defect distribution, by constructing a convolutional model of the ultrasonic TOFD signal, the Orthogonal Matching Pursuit (OMP) sparse deconvolution algorithm is utilized to enhance the longitudinal resolution. Based on the synthetic aperture acoustic imaging model, in the wavenumber domain, backpropagation inference is implemented through phase transfer technology to eliminate the influence of diffraction effects and enhance transverse resolution. On this basis, the time-domain sparse deconvolution and frequency-domain synthetic aperture focusing methods mentioned above are combined to enhance the resolution of ultrasonic TOFD imaging. The simulation and experimental results indicate that this technique can outline the shape of defects with fine detail and improve image resolution by about 35%.

## 1. Introduction

Ultrasonic TOFD imaging, as an important imaging method for pipeline girth weld nondestructive testing, uses diffraction time difference as the basic parameter for quantitatively detecting internal defects. It complements the phased array zonal ultrasonic imaging method and can provide a more precise defect distribution of girth welds. TOFD has become one of the most important methods for the quantitative evaluation of weld quality, especially for the quantitative characterization of the size and burial depth of vertical cracks inside the weld seam, as it exhibits obvious technical advantages. Meanwhile, TOFD is a low-cost method for weld inspection. The number of probes and data acquisition channels required is significantly lower than that of phased array equipment. However, this imaging method has always been affected by signal oscillations caused by the limited frequency band of the transducer and the inherent diffraction effect of defective echo scattering, resulting in unsatisfactory longitudinal and transverse resolutions, the presence of detection blind areas, and insufficient detection accuracy. In addition, the real-time limitations of its time-domain imaging method seriously affect its detection performance and online application ability. For this reason, classic deconvolution techniques have been applied to improve the longitudinal resolution of ultrasonic imaging. Parameterized inverse filtering methods [1,2] that regard deconvolution as an inverse filtering process have excellent flexibility. The imaging effect strongly relies on the correct setting of weight parameters, which can be estimated from the ultrasonic image itself. In most cases of pipeline weld inspection, the defect information is sparse and not conducive to the estimation of weight parameters. High-order spectral analysis (cepstral theory) [3,4] is based on energy spectrum analysis, which has the advantage of high computational efficiency. Such a method is not well suited for non-stationary signals like ultrasonic TOFD signals. L2 regularized norm reconstruction [5,6] takes the deconvolution problem as a linear optimization process and optimizes it with the L2 norm as the constraint term. L2 is a smoothing constraint, and it is still difficult to meet the demand for longitudinal resolution optimization. Besides the above, wavelet transformation is also utilized to improve the image quality of TOFD [7,8]. It can reduce the noise level and improve the longitudinal resolution by combining it with the match pursuit process [9]. Nevertheless, there are still technical challenges, such as a low contrast ratio and the need for further resolution enhancement.

To improve lateral resolution, the time-domain SAFT is mainly used [10,11], which calculates the wave arrival delay between the transducer and the defect and reconstructs the defect image data through the Delay-and-Sum (DAS) rule. In [12], a time-domain SAFT integrated deconvolution was studied, improving both the transverse and longitudinal resolutions for a point defect. Although the time-domain SAFT has simple calculations, it requires point-to-point calculations of the imaging area, which limits its efficiency. An alternative solution is to reconstruct the image using machine learning methods [13,14]. Such a solution can achieve excellent performance in trained scenarios but requires more samples and training resources for other cases. Recently, frequency-domain SAFTs, also known as wavenumber-domain SAFTs, have been widely studied due to their high-quality imaging results and high computational efficiency. The performance of frequency-domain SAFTs has been verified through different modes, such as monostatic ultrasonic imaging [15,16], total focusing method ultrasonic imaging [17,18], and compound plane-wave ultrasonic imaging [19,20]. However, these methods rely on specific ultrasonic imaging models that have not yet been explored in TOFD ultrasonic imaging.

Based on the above, we study online high-resolution ultrasonic TOFD imaging technology for pipeline girth welds. The proposed method is the first to report the frequency-domain SAFT for ultrasonic TOFD, which integrates sparse deconvolution to achieve improvements in both longitudinal and transverse resolutions. Meanwhile, the processing has extremely low time costs, providing the potential for real-time quantitative TOFD detection. According to the simulation and experimental results, the proposed method can outline the shape of defects with very fine detail, and the transverse resolution of the image improves by about 35%. Also, the processing time of the proposed method is 0.96 s, which is much shorter than the TOFD data acquisition time, showing the potential for real-time inspection.

## 2. Theoretical Expression of the General Model for Ultrasonic TOFD Imaging Detection Signals

**Figure 1:**
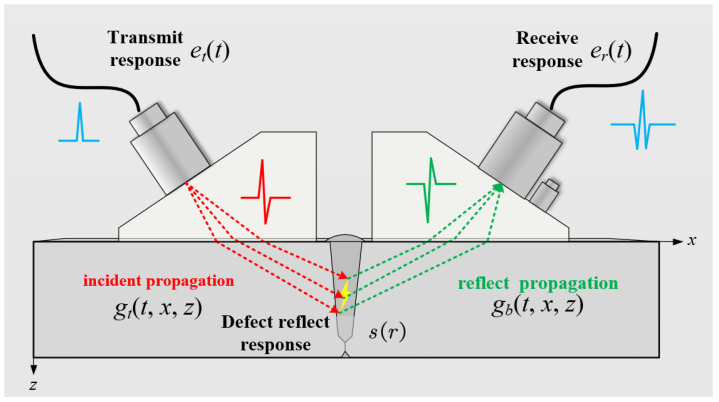
The propagation process of ultrasonic waves in TOFD imaging.

Ultrasonic TOFD imaging mainly involves five stages from emission to reception: transducer excitation, incident propagation, defect response, reflection propagation, and transducer reception, as shown in Figure 1. According to classical linear system theory, the general theoretical expression for the ultrasonic TOFD detection signal is

(1) $$y\left(\hat{x},t\right)=e_{t}\left(t\right)⨂\left[\;\int \int \;g_{t}\left(t,x,z\right)⨂s\left(x,z\right)⨂g_{b}\left(t,x,z\right)dxdz\right]⨂e_{r}\left(t\right)$$

Among them, $y(\hat{x},t)$ represents the received TOFD signal; $e_{t}\left(t\right)$ and $e_{r}\left(t\right)$ represent the excitation response function and the reception response function of the ultrasonic transducers, respectively; $g_{t}\left(t,x,z\right)$ and $g_{b}\left(t,x,z\right)$ represent the spatiotemporal response functions of the incident propagation path and the received propagation path; s(x,z) represents the spatial distribution function, which characterizes the reflection response of the defect; $\hat{x}$ represents the detection position of the TOFD signal; and *x* and *z* denote the spatial coordinates of a specific point in the ROI. s(x,z) is also the reconstruction goal of high-resolution ultrasonic TOFD imaging, and ⨂ represents the convolution operator.

Although all five stages mentioned above are correlated using convolution, their convolution dimensions and methods are different. $e_{t}\left(t\right)$ and $e_{r}\left(t\right)$ modulate signals in the time domain, and their convolution occurs in the time domain, mainly manifested in their effect on signal bandwidth. However, $g_{t}\left(t,x,z\right)$ and $g_{b}\left(t,x,z\right)$ are convolutions in the spatiotemporal domain and mainly represent the diffraction effect of ultrasonic waves in physical space. Furthermore, Equation (1) can be separated into two separate parts, and it is expressed in the spatial frequency domain as

(2) $$y\left(\hat{x},\omega \right)=\left[e_{t}\left(\omega \right)\cdot e_{r}\left(\omega \right)\right]\cdot p\left(\hat{x},\omega \right)$$

(3) $$p\left(\hat{x},\omega \right)=\;\int \int \;G_{t}\left(\omega ,x-\hat{x},z\right)\cdot s\left(x,z\right)\cdot G_{b}\left(\omega ,x-\hat{x},z\right)dxdz$$

As shown in Equation (2), the reflection coefficient function $p(\hat{x},\omega )$ is extracted as an intermediate term. The above two equations are the two problems addressed in this paper, where $p(\hat{x},\omega )$ is calculated from the measured data $y(\hat{x},\omega )$ to mitigate signal bandwidth degradation caused by the transducer response and improve the longitudinal resolution of TOFD imaging. In contrast, using $p(\hat{x},\omega )$ to solve for s(x,z) eliminates diffraction effects and improves the lateral resolution of TOFD imaging. Of course, the exact form of the solution will be adapted to the specific situation, but fundamentally, it enhances image resolution both temporally and spatially by addressing signal bandwidth limitations and diffraction effects.

## 3. High-Longitudinal-Resolution Ultrasonic TOFD Imaging Based on Sparse Deconvolution

### 3.1. Sparse Deconvolution Model for Ultrasonic TOFD Signals

Due to the limitations of the bandwidth of the transducer, the transmitted and received ultrasonic waves have multiple periodic oscillations, resulting in a decrease in the longitudinal (i.e., depth) resolution of TOFD imaging. These periodic oscillations can be modeled as the convolution of the transducer’s emitted and received electronic pulse responses, which are irrelevant to physical space. The effect of spatial dimensions can be ignored when eliminating transducer response effectiveness. At this point, $p(\hat{x},\omega )$ and $y(\hat{x},\omega )$ can be approximated as p(ω) and y(ω). Here, we use the time-domain one-dimensional discrete convolutional model to represent Equation (2) in the time domain:

(4) y=e⨂p+n

In this equation, $y={\left[y\left(0\right)\;y\left(1\right),\ldots ,y\left(M-1\right)\right]}^{T}$ represents the received TOFD signal, and $e={\left[e\left(0\right)\;e\left(1\right),\ldots ,e\left(M-1\right)\right]}^{T}$ represents the overall response of the transducer’s emitted and received electronic pulses, which can be expressed as $e\left(t\right)=e_{t}\left(t\right)⨂e_{r}\left(t\right)$. $p={\left[p\left(0\right)\;p\left(1\right),\ldots ,p\left(M-1\right)\right]}^{T}$ represents the reflection coefficient function of the acoustic reflector, and $n={\left[n\left(0\right)\;n\left(1\right),\ldots ,n\left(M-1\right)\right]}^{T}$ represents the noise. It should be noted that the above convolution operation constructs the incident reference signal with zero as the center reference point and intercepts the first M vector dimensions after convolution as the representation of signal y. Therefore, the vector dimensions remain consistent before and after the convolution operation. Equation (4) is a standard linear model of ultrasonic imaging. Since only defects reflect ultrasonic waves in the TOFD imaging system, the reflection coefficient function p can be used to describe defect characteristics. This linear model is also used in different ultrasonic imaging techniques [21,22,23]. The electronic pulse response e can be established through the testing frequency response characteristics of various electronic devices, or a set of large plane ultrasonic echo signals can be directly selected for measurement because the large plane echo signals already approximate the information of ultrasonic signal periodic oscillations.

Obviously, the reflection coefficient function p contains all the defect information of the object to be tested and is not limited by the ultrasonic signal bandwidth, achieving extremely high longitudinal resolution. Therefore, it is necessary to obtain the reflection coefficient function p of the object to be tested and solve it using Equation (4) with known y and e. This process is called deconvolution. Due to the presence of noise in the actual signals, there is not a strict convolutional relationship between the TOFD signal, the electronic pulse response, and the reflection coefficient function. Directly performing deconvolution can easily lead to an ill-conditioned solution. To mitigate the impact of noise on the solution, the deconvolution process is transformed into a linear equation-solving approach. To construct the linear equation corresponding to Equation (4), the Fourier transform matrix is introduced to perform the Fourier transform:

(5) E=Fx

In this equation, E is defined as the spectrum of the electronic pulse response, while the Fourier transform matrix F is defined as

(6) $$F=\left[\begin{array}{cccc} 1 & 1 & ... & 1\\ 1 & \omega & ... & 1\\ . & . & . & .\\ . & . & . & .\\ . & . & . & .\\ 1 & \omega^{M-1} & ... & \omega^{\left(M-1)(M-1\right)} \end{array}\right],\;\;\;\;\omega =e^{-j2\pi /M}$$

Then, by diagonalizing the frequency spectrum E of the electronic pulse response, a Toeplitz cyclic matrix C can be constructed as follows:

(7) $$C=\frac{1}{M}F^{†}diag\left(E\right)F$$

In this equation, $\frac{1}{M}$ is used to balance the energy changes during discrete Fourier calculations, and † is a conjugate operator. The Toeplitz cycle matrix has generalized diagonality, which can be seen as the spatial translation of the electronic pulse response e in the matrix C. Obviously, multiplying vector p by matrix C yields the same result as performing truncated and equal-length sum convolution [6]. Therefore, the linear equation of Equation (4) is

(8) y=Cp+n

In this equation, C is a square matrix $R^{M\times M}$. According to the least squares method, the solution of Equation (5) can be expressed as

(9) $$\min_{p}||y-{Cp||}_{2}^{2}$$

where $\left|\right|\cdot {\left|\right|}_{2}^{2}$ represents the $l_{2}$ norm.

Theoretically, when the noise level is not high or the condition number of the convolutional matrix C is not large, using the least squares method can partly suppress noise and solve for the reflection coefficient function p. A low noise level here mainly corresponds to a signal-to-noise ratio greater than 0 dB, and often higher than 12 dB. In practical applications, the condition number of the convolutional matrix C can be approximately interpreted as the ultimate expected resolution limit of the electronic pulse response. When constructing the convolutional matrix C, the size of the translation interval depends on the ultimate resolution limit. When the translation interval increases and the correlation between row and column vectors decreases to half of their own energy, the condition number satisfies the requirement for stable solvability. However, in practical applications, if the translation interval directly follows the discrete sampling interval, the correlation between row and column vectors will be much greater than half of their own energy, leading to an ill-conditioned equation. Meanwhile, Equation (9) uses the $l_{2}$ norm as the optimization objective, which is similar to a Gaussian distribution, resulting in excessively smooth solutions and limited improvement in longitudinal resolution.

Usually, in the application environment of ultrasonic non-destructive testing, the probability of defects appearing is relatively low, and their distribution exhibits high sparsity; that is, the proportion of defect areas in the tested object is very small, independent, and discrete. Therefore, a prior assumption of sparse distribution can be introduced into the reflection coefficient function p of the tested object by adding a set of sparse constraints to the above optimization objective function, rewriting it as

(10) $$\min_{p}||y-{Cp||}_{2}^{2}{\;\;\;s.t.\;min||p||}_{0}$$

The $l_{0}$ norm in the above formula represents the number of nonzero elements in p, and this type of constraint applied to the model is called regularization. Common regularization methods include $l_{2}$ constraints (Tikhonov regularization constraints) and $l_{1}$ constraints. The $l_{0}$ constraint term added strongly enforces sparsity in p while trying to avoid ill-conditioned solutions caused by instability in the equation-solving process. While correctly identifying defect positions, it can effectively suppress the smoothing effect introduced by the $l_{2}$ norm in Equation (10) and improve the longitudinal resolution of ultrasonic TOFD imaging as much as possible.

### 3.2. Sparse Deconvolution Optimization Method for Ultrasonic TOFD Signals

Based on the linear equation model in Equation (10), multiple optimization methods can be used to solve it, which include common algorithms such as base pursuit (BP) [15,16,17] and matching pursuit (MP) [18,19,20], but their solution approaches are vastly different. The BP algorithm can be seen as a subtraction operation, which projects the echo signal into a new space through the inverse mapping of the matrix C. In this space, the defect energy is concentrated and the amplitude is amplified, while the noise energy is dispersed and the amplitude is reduced. Therefore, during the iterative process of solving, the main energy is retained by gradually eliminating the excess small amplitude components, thus achieving the goal of sparse reconstruction. Since the BP calculation process requires the Jacobian or Hessian matrix of linear equations, the solving process still relies on convex optimization theory, which imposes certain limitations on the model and is not compatible with $l_{0}$ norm-constrained problems. MP-class methods are mainly based on greedy algorithms [21], similar to incremental operations. After initialization, they are set to zero vectors and continuously add non-zero terms during iteration to approximate the optimization results. Compared to other methods, for ultrasonic TOFD detection, solving targets based on $l_{0}$ norm constraints is better suited for optimization using MP-class algorithms. Thus, the Orthogonal Matching Pursuit (OMP) algorithm [22] is used to solve Equation (7). Before introducing the OMP algorithm, the conventional MP algorithm is introduced. Its main process is as follows:

STEP 1: establish dictionary codebook D, initialize atomic library B)={ϕ}, residual R)=y), sparsity *K*, and iterative counter k=0;

STEP 2: if k⩽K

➀: calculate degree of relevance: $T^{k}=D^{k}R^{k}$

➁: select the optimal atom: $B^{k}=\left\{B^{k},max\left(T_{i}^{k}\right)\right\}$

➂: update residuals: $R^{k}=y-D^{T}B^{k}$

➃: next: k=k+1, go to STEP 2;

else goto STEP 3;

STEP 3: end the loop and obtain the result: $p=B^{k}$.

In the initialization process, the dictionary codebook D is a sparse spatial mapping matrix. In ultrasonic non-destructive testing, the reflection coefficient function p exhibits sparsity, and the matrix C exhibits cyclic traversal characteristics. Therefore, the codebook D can be directly replaced with convolutional matrices C. In correlation calculation, the atom with the highest correlation is calculated by the inner product between the estimated residuals and the codebook. Then, the atom with the highest degree of relevance is selected and added to the excitation atom library. Meanwhile, the residual value is updated, and this cycle repeats until the sparsity counter is satisfied.

The dictionary codebook C mentioned here is actually a set of bases. If this set of bases Φ is orthogonal, then

(11) $$T=\Phi^{T}y=\Phi^{T}\Phi p$$

It can be seen that when the codebook is an orthogonal basis, T can be directly mapped to p. Therefore, it is reasonable to represent the main sparse components by selecting several points with the highest degree of relevance in the vector T. Typically, the codebooks D are redundant bases, and their degree of redundancy depends on the condition number of the convolutional matrix C. This leads to a non-linear mapping relationship between T and p. To eliminate the difference between T and p caused by the correlation between redundant bases, it is necessary to orthogonalize the matching pursuit using the OMP (Orthogonal Matching Pursuit) method [24,25].

The orthogonalization of OMP is achieved by eliminating the correlation between vectors within redundant bases, achieving more accurate matching. The basic process of OMP is similar to that of MP, with slight differences in the correlation calculation [26]. Assuming there are already *k* atoms, and the normalized basis vectors corresponding to these excited atoms in the codebook are denoted as $\left\{d^{1},d^{2},...,d^{k}\right\}$. In order to eliminate the impact of the non-orthogonality of each vector on the redundant bases, the new normalized basis vector is modified during the next round of correlation calculation:

(12) $$d^{k+1}=d_{o}^{k+1}-\sum_{i=1,2,\ldots ,k}d^{i}<d_{o}^{k+1},d^{i}>$$

where $d_{o}^{k+1}$ is the normalized basis vector corresponding to the selected atom in the next iteration in Equation (12). Obviously, Equation (12) ensures that the normalized vector is continuously reconstructed during the iteration process, forming an effect similar to orthogonal bases to maximize the accuracy of sparse imaging as much as possible.

The OMP calculation has a hyperparameter, sparsity *K*, and the selection of sparsity *K* should be based on the specific ultrasonic testing application. For ultrasonic TOFD, each A-scan signal contains a lateral wave and a backwall reflection wave, so at least two atoms in B need to be represented. In the presence of defects, there are also diffraction waves at the defect tips. Due to the independent and discrete sparse distribution of defects, it is assumed that only one set of defects exists in a single A-scan signal at a time. Therefore, setting the value of *K* to 3 or 4 is more appropriate. It should be noted that in subsequent experiments, the lateral wave is truncated and omitted after straightening, so its sparsity *K* is correspondingly reduced by 1.

## 4. High-Transverse-Resolution Ultrasonic TOFD Imaging Based on Frequency-Domain SAFT

The scanning imaging process of ultrasonic TOFD for pipeline girth welds is essentially a D-scan that moves along the girth direction. The transducers are symmetrically positioned on both sides of the weld seam and driven by a mechanical device. In an ideal situation, when the acoustic axis of the transmitting transducer passes through a defect, an extremely strong reflected echo will be generated and captured by the receiving transducer for detecting the target defect. However, in practical applications of TOFD, the directionality of sound waves differs from that of lasers; instead, they generate diffraction at the defect tips, forming arc-shaped reflected waves. Therefore, even point defects can be detected by probes at multiple locations, forming a “tail”-shaped, curved defect image. For continuous long-strip cracks, there are obvious trailing arcs at both ends of the crack, which can easily lead to inaccurate assessments of defect size and burial depth.

To address the problem of the reduced transverse resolution mentioned above, it is often necessary to use the synthetic aperture focusing technique (SAFT) [21,27] to reconstruct images. However, the D-scan image of TOFD differs from conventional two-dimensional B-scan images; its essence is to project three-dimensional spatial information into two-dimensional images, which inevitably leads to certain information losses. The most direct example is that the D-scan only collects one A-scan line per axial section and estimates the defect position based on the time delay of the reference echo. However, for the same echo time delay, there are multiple possible defect positions on its axial section, and their specific positions cannot be determined. Usually, the SAFT for TOFD imaging follows the central imaging assumption, assuming that defects are located at the centerline of the transducer pair. When a defect is assumed to fall on the centerline of the transducer pair, the emission and reception of sound waves naturally satisfy the condition of a symmetrical sound path; that is, the emission path and reception path are symmetrical about the centerline.

According to Equation (1), the spatial diffraction propagation process of ultrasonic TOFD can also be constructed using a convolutional model, which is usually defined in the analytical model as a finite amplitude delta signal with amplitude information s(x,z). As shown in Equation (3), the convolution model can be simplified by multiplying the transmitting spatial-frequency function $G_{t}\left(\omega ,x-\hat{x},z\right)$, the amplitude information s(x,z), and the receiving spatial-frequency function $G_{b}\left(\omega ,x-\hat{x},z\right)$ in the frequency domain. In order to associate the angular frequency ω with the propagation direction of ultrasonic waves, ω is replaced with the wavenumber k=ω/c. Thus, Equation (3) is rewritten as

(13) $$p\left(\hat{x},k\right)=\;\int \int \;G_{t}\left(k,x-\hat{x},z\right)\cdot s\left(x,z\right)\cdot G_{b}\left(k,x-\hat{x},z\right)dxdz$$

Image reconstruction is the inverse computational process of s(x,z) from p(x,k). Neglecting the multiple reflections of sound waves during propagation, the transceiver path is symmetrical. Thus, the spatial-frequency functions for transmitting and receiving are equivalent:

(14) $$G\left(k,x-\hat{x},z\right)=G_{t}\left(k,x-\hat{x},z\right)=G_{b}\left(k,x-\hat{x},z\right)$$

Since $G(k,x-\hat{x},z)$ can be regarded as the 2D free-space Green’s function, given by $G\left(k,x-\hat{x},z\right)=\exp\left(ik\sqrt{\left(x-\hat{x}\right)+z^{2}}\right)/\sqrt{\left(x-\hat{x}\right)+z^{2}}$, Equation (13) can be rewritten as

(15) $$\begin{array}{cc} \; & p\left(\hat{x},k\right)=\;\int \int \;\frac{\exp\left(2\cdot ik^{\prime }\sqrt{{\left(x-\hat{x}\right)}^{2}+z^{2}}\right)}{\sqrt{{\left(x-\hat{x}\right)}^{2}+z^{2}}}\cdot s\left(x,z\right)dxdz\\ \; & k^{\prime }=2k=\frac{2\omega }{c}=\frac{\omega }{c_{erm}} \end{array}$$

In TOFD, the transmitting and receiving probes are symmetrically positioned on both sides of the weld. Therefore, the processes of wave incidence onto central weld defects, wave scattering from defects, and their reception by the receiver are also symmetrical. To simplify the calculation of symmetrical bidirectional wave field propagation, the Explosion Reflection Model (ERM) is introduced. Under the ERM, we assume that the sound speed of the propagating medium is half of its real value (cerm = c/2), and the propagation process can be equivalently modeled as ultrasonic waves being autonomously emitted from each pixel point, which is then received by the ultrasonic transducer. The phase information of the sound waves is consistent with the self-emission and reception scenario, and the waveform remains unchanged along the time axis. In this model, all scatter points are set to emit sound waves at the same time—referred to as the “explosion” moment—and their intensity is directly proportional to the reflection coefficient of each point. The sound fields produced by these points are emitted and received simultaneously by the ultrasonic transducer. This simplifies the bidirectional wave propagation and only considers the sound field received from the upward returning probe.

According to Weyl’s integral, the Green’s function in 2D free space can be expanded and expressed as

(16) $$\frac{\exp\left(2\cdot ik^{\prime }\sqrt{{\left(x-\hat{x}\right)}^{2}+z^{2}}\right)}{\sqrt{{\left(x-\hat{x}\right)}^{2}+z^{2}}}=\frac{-i}{4\pi }\int \frac{\exp\left(ik_{x}\left(x-\hat{x}\right)-i\left|z\right|\sqrt{k^{\prime 2}-k_{x}^{2}}\right)}{\sqrt{k^{\prime 2}-k_{x}^{2}}}dk_{x}$$

Substituting Equation (16) into Equation (15) yields

(17) $$p\left(\hat{x},k^{\prime }\right)=\frac{-i}{4\pi }\int \frac{\exp\left(ik_{\hat{x}}\hat{x}\right)}{\sqrt{k^{\prime 2}-k_{\hat{x}}^{2}}}\left(\;\int \int \;\exp\left(-ik_{\hat{x}}x-i\left|z\right|\sqrt{k^{\prime 2}-k_{\hat{x}}^{2}}\right)\cdot s\left(x,z\right)dxdz\right)dk_{\hat{x}}$$

It can be seen that the relationship between $p(\hat{x},k)$ and s(x,z) established by Equation (17) has a complex Fourier decomposition relationship, and an inverse Fourier transform is performed over $k^{\prime }$. Equation (17) can be rewritten as

(18) $$p\left(k_{\hat{x}},k^{\prime }\right)=\frac{-i}{4\pi }\cdot \frac{1}{\sqrt{k^{\prime 2}-k_{\hat{x}}^{2}}}\left(\;\int \int \;\exp\left(-ik_{\hat{x}}x-i\left|z\right|\sqrt{k^{\prime 2}-k_{\hat{x}}^{2}}\right)\cdot s\left(x,z\right)dxdz\right)$$

For convenience, we assume that $k_{x}=k_{\hat{x}}$ when the size of the ROI remains unchanged in the depth direction. Similarly, the amplitude information s(x,z) can be replaced with the spectrum $S\left(k_{x},z\right)$ through a Fourier transform over *x*

(19) $$P\left(k_{\hat{x}},k^{\prime }\right)=\frac{-i}{4\pi }\cdot \frac{1}{\sqrt{k^{\prime 2}-k_{\hat{x}}^{2}}}\int \exp\left(-i|z|\sqrt{k^{\prime 2}-k_{x}^{2}}\right)\cdot S\left(k_{x},z\right)dz$$

According to the analysis based on the plane-wave model, the acoustic phase $\exp\left(-i\left|z\right|\sqrt{k^{\prime 2}-k_{x}^{2}}\right)$ has a significantly greater modulation effect on the signal than $\sqrt{k^{\prime 2}-k_{\hat{x}}^{2}}$; thus, $\sqrt{k^{\prime 2}-k_{\hat{x}}^{2}}$ can be neglected. Since the final image can be normalized, the constant coefficient $-i/\left(4\pi \right)$ is also discarded. Equation (19) is simplified to

(20) $$P\left(k_{x},k^{\prime }\right)=\int \exp\left(-i|z|\sqrt{k^{\prime 2}-k_{x}^{2}}\right)\cdot S\left(k_{x},z\right)dz$$

Equation (20) can be rewritten as the recursive formula

(21) $$\begin{array}{cc} \; & S\left(k_{x},z\right)=P\left(k_{x},k^{\prime }\right)\exp\left(ik_{z}z\right)\\ \; & k_{z}=\sqrt{k^{\prime 2}-k_{x}^{2}} \end{array}$$

where $k_{z}$ is the wavenumber along the z-axis. The whole process of wavefield derivation involves calculating $S\left(k_{x},z\right)$ and thus estimating the defect signal s(x,z) with the given wavefield $P\left(k_{x},k^{\prime }\right)$. However, unlike the conventional B-scan, the deduced depth information *z* of the D-scan is not directly equal to the defect depth. Because of the lateral distance PCS between the transducer’s scanning plane and the imaging plane, the deduced depth should be corrected as follows:

(22) $$z^{\prime }=\sqrt{PCS^{2}+z^{2}}$$

The corresponding derivation formula is given by

(23) $$S\left(k_{x},z\right)=P\left(k_{x},k^{\prime }\right)\exp\left(ik_{z}\sqrt{PCS^{2}+z^{2}}\right)$$

The amplitude information s(x,z) is calculated by performing an inverse Fourier transform over $k_{x}$. Since the actual signals are band-limited, the final image is obtained by superimposing all $\left(k_{x},z\right)$ values over the wavenumber $k^{\prime }$ as follows:

(24) $$s\left(x,z\right)=\;\int \int \;S\left(k_{x},z\right)\exp\left(ik_{x}x\right)dk_{x}dk^{\prime }$$

In summary, the high lateral resolution is obtained with the following calculation steps.

The longitudinal-resolution-optimized data p are written as $\left[p_{1},p_{2},\ldots ,p_{K}\right]$, where $p_{k}$ is given by $p\left(k,\omega \right)={\left[p_{k}\left(0\right),p_{k}\left(0\right),\ldots ,p_{k}\left(M-1\right)\right]}^{T}$. $p_{k}$ is calculated using the sparse deconvolution for each position as

(25) $$\begin{array}{c} \min_{p_{k}}{∥y_{k}-C_{k}p_{k}∥}_{2}^{2}\;\;s.t.\min{∥p_{k}∥}_{0} \end{array}$$

The Fourier transform matrix $F_{k\hat{x}}$ over $\hat{x}$ is defined as

(26) $$F_{k\hat{x}}=\left[\begin{array}{cccc} 1 & 1 & \ldots & 1\\ 1 & k_{\hat{x}} & \ldots & 1\\ . & . & . & .\\ . & . & . & .\\ . & . & . & .\\ 1 & {k_{\hat{x}}}^{K-1} & \ldots & {k_{\hat{x}}}^{\left(K-1)(K-1\right)} \end{array}\right],\;k_{\hat{x}}=e^{-i2\pi /K}$$

The following recursive wavefield derivation at depth *z* involves left-multiplying the dispersion relation matrix Z:

(27) $$\begin{array}{cc} \; & Z=\left[\begin{array}{cccc} k_{z}\left(0,0\right) & k_{z}\left(0,1\right) & \ldots & k_{z}\left(0,M-1\right)\\ k_{z}\left(1,0\right) & k_{z}\left(1,1\right) & \ldots & k_{z}\left(1,M-1\right)\\ . & . & . & .\\ . & . & . & .\\ . & . & . & .\\ k_{z}\left(K-1,0\right) & k_{z}\left(K-1,1\right) & \ldots & k_{z}\left(K-1,M-1\right) \end{array}\right],\\ \; & \;\;k_{z}\left(k,m\right)=\exp\left\{i\sqrt{\frac{{\left[\frac{2\pi }{M\Delta t}\cdot \left(-\frac{M}{2}+m\right)\right]}^{2}}{c_{erm}^{2}}-{\left[\frac{2\pi }{K\Delta x}\cdot \left(-\frac{K}{2}+k\right)\right]}^{2}}\sqrt{PCS^{2}+z^{2}}\right\} \end{array}$$

The inverse Fourier transform matrix $F_{x}$ over $k_{x}$ is similarly defined as

(28) $$F_{x}=\left[\begin{array}{cccc} 1 & 1 & \ldots & 1\\ 1 & x & \ldots & 1\\ . & . & . & .\\ . & . & . & .\\ . & . & . & .\\ 1 & x^{K-1} & \ldots & x^{\left(K-1)(K-1\right)} \end{array}\right],\;x=e^{-i2\pi /K}$$

The superimposition over ω involves right-multiplying the constant matrix E as

(29) $$E=\left[\begin{array}{c} 1\\ 1\\ .\\ .\\ .\\ 1 \end{array}\right]$$

The final image $I_{z}$ at depth *z* is calculated using the aforementioned matrices as

(30) $$I_{z}=\left\{F_{x}\left[Z⊙\left(F_{k\hat{x}}p\right)\right]\right\}E$$

where ⊙ represents the Hadamard matrix multiplication. The wavefield calculation for $I_{z}$ is then performed for each depth.

**Figure 2:**
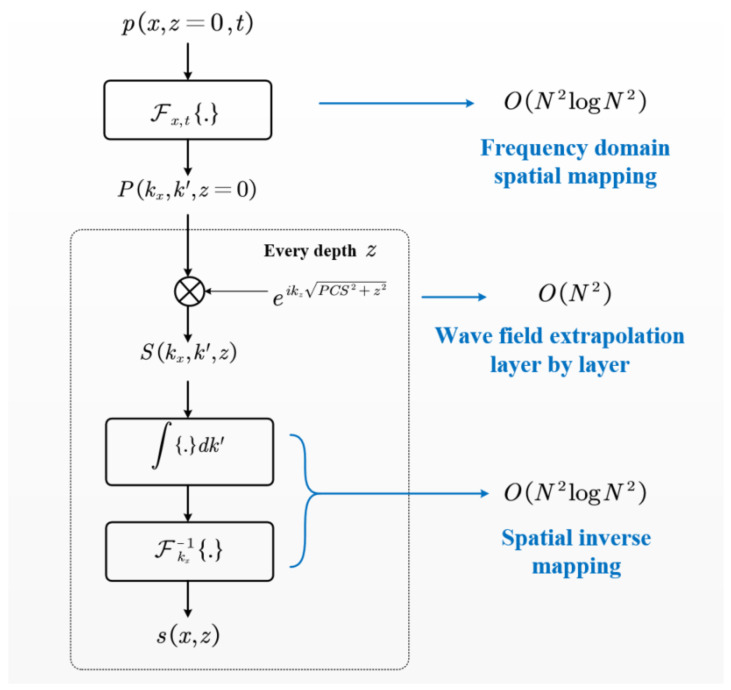
Frequency-domain SAFT reconstruction process for TOFD D-scan images and its computational complexity at each step.

The entire computational workflow, depicted in Figure 2, primarily consists of three steps: frequency-domain spatial mapping, phase migration, and imaging spatial inverse mapping. In the frequency-domain spatial mapping step, a two-dimensional Fourier transform is employed to map the received signal $p(\hat{x},t)$ to $P\left(k_{\hat{x}},k^{\prime }\right)$, with a computational complexity of approximately $O(N^{2}\log N^{2})$. The phase migration step involves extrapolating the mapped wavefield layer by layer to gather acoustic field information at different depths. The core computational step of this process is accomplished using the Hadamard product, resulting in a complexity of $O(N^{2})$. The overall computational complexity is $O(N^{3})$ for the case of *N* imaging depth layers. The final step, imaging spatial inverse mapping, is conducted through the imaging condition formula, with a complexity of $O(N^{2}\log N^{2})$. When combining these three steps, the computational complexity of the entire image reconstruction is $O(N^{3})$, which is evidently more efficient than the $O(N^{4})$ required by the traditional SAFT.

## 5. Ultrasonic TOFD Imaging with Both High Longitudinal and Lateral Resolutions

**Figure 3:**
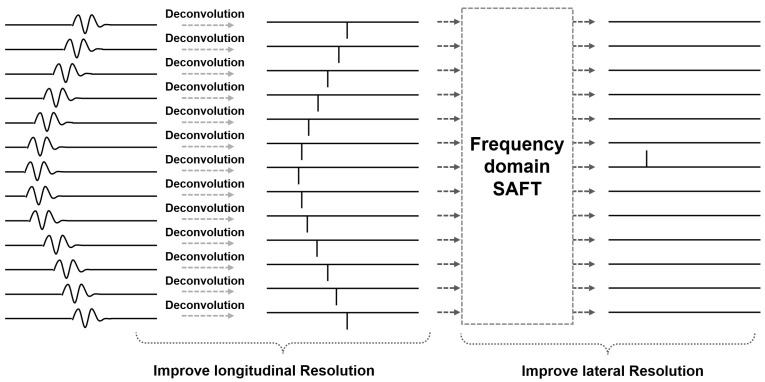
The image reconstruction process of the proposed method.

Since the process of improving the longitudinal and lateral resolutions is holistic, we unify the two procedures. The acquired raw data y are denoted as $\left[y_{1},y_{2},\ldots ,y_{K}\right]$, where $y_{k}$ is equal to $y\left(k,\omega \right)={\left[y_{k}\left(0\right),y_{k}\left(0\right),\cdots ,y_{k}\left(M-1\right)\right]}^{T}$. First, the optimized data p containing all the scanning positions are calculated in order to improve the longitudinal resolution. Second, the lateral resolution is improved using the frequency-domain SAFT, and the final image is then retrieved. The process can be written as

(31) $$\begin{array}{cc} \; & I_{z}=\left\{F_{x}\left\{Z⊙\left[F_{k\hat{x}}\left(\min_{p}{∥y-\mathrm{Cp}∥}_{2}^{2}\;\;s.t.\min{∥p∥}_{0}\right)\right]\right\}\right\}E\\ \; & I=\left[I_{1},I_{2},\cdots ,I_{M}\right] \end{array}$$

where *M* represents the maximum depth in the final image. The image reconstruction process of the proposed method is illustrated in Figure 3. It should be noted that the proposed method is established on the hypothesis of known sound velocity. Also, all the calculations are performed for single-channel TOFD imaging.

## 6. Simulation and Experimental Studies

**Figure 4:**
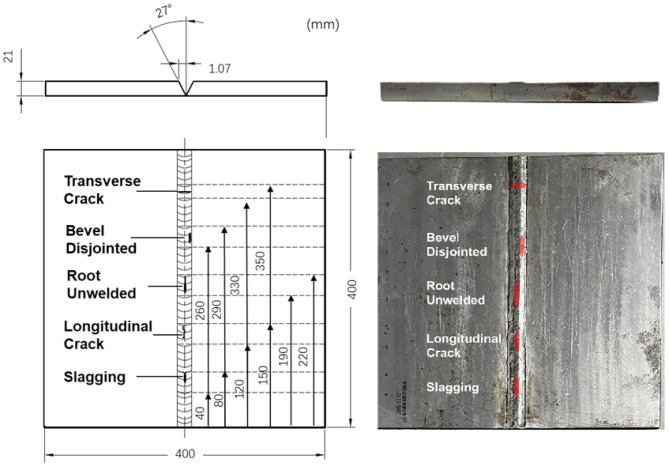
V-bevel weld specimen with artificial defects.

In order to verify the effectiveness of the above method, simulation and experimental studies were carried out, and the design drawings and physical photographs of the specimen used are shown in Figure 4. The specimen is a flat plate that is butt-welded, and the inside of the weld contains five types of defects, including open transverse cracks, unfused bevels, root under-welding, longitudinal cracks, and slag entrapment. The thickness of the plate is 21 mm.

### 6.1. Simulation

**Figure 5:**
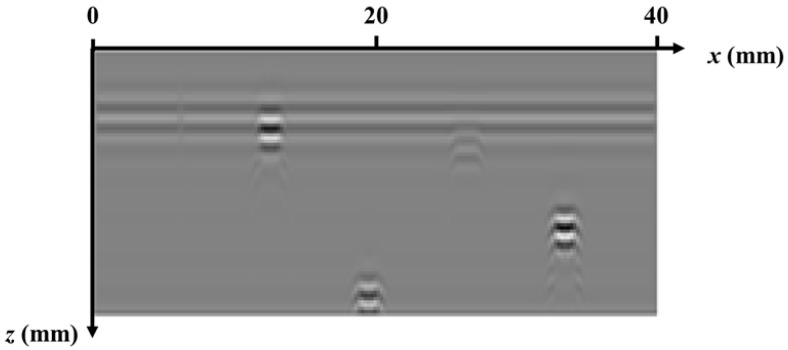
Simulated images generated from raw data.

The data generation for the simulation is based on the simulation software CIVA 2020. A transceiver TOFD transducer with a diameter of 6 mm, a center frequency of 5 MHz, and a bandwidth of 70% (−6 dB) is selected. The wedge refracting angle is 60°, the Probe Center Separation (PCS) is 48.5 mm, the scanning interval is 0.5 mm, and the transmitting transducer and receiving transducer are symmetrically arranged on both sides of the weld. At each scanning position, 10 μs of data are recorded with a sampling rate of 50 MHz, and as a result, each A-scan line contains 500 data points. The whole scanning process involves 80 steps. The collected simulation data form an 80 × 500 matrix and generate the D-scan TOFD image shown in Figure 5.

**Figure 6:**
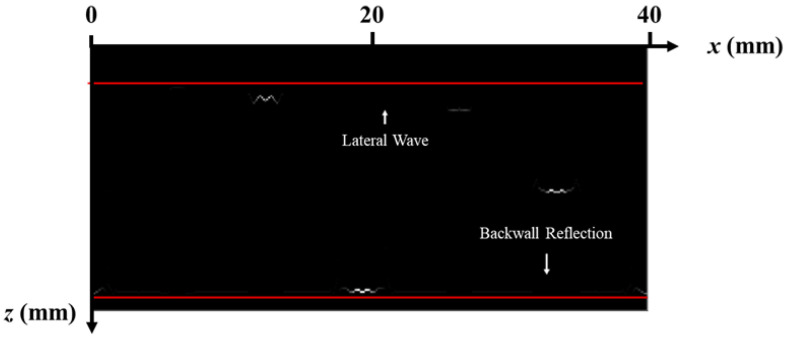
Reconstruction results of the frequency-domain synthetic aperture and inverse convolution fusion method proposed in this paper.

Due to the diffraction effect of ultrasonic waves, the tips of defects present arc-shaped artifacts, which can easily cause errors in the judgment of defect size and burial depth. For this reason, the proposed frequency-domain synthetic aperture and sparse deconvolution methods are fused to achieve the acoustic reconstruction of the D-scan TOFD imaging, and the results are shown in Figure 6. The figure shows that the spatial resolution of ultrasonic TOFD imaging is greatly improved, which lays a good foundation for the quantitative detection of annular weld defects in terms of size and embedment depth. However, it should be noted that weaker transverse crack signals are easily rejected after the above processing.

### 6.2. Experimental Studies

**Figure 7:**
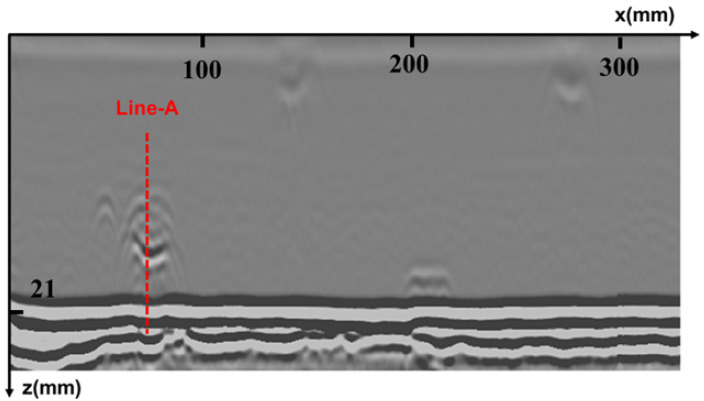
Raw data imaging results.

The specimen used in the experiment is consistent with that used in the simulation, with a V-type groove welded by argon-arc and manual electric-arc welding modes, and five sets of artificial defects embedded in the same manner as in the simulation. The TOFD transducers are the same as those used in the simulation, and the PCS is 48.5 mm. These two transducers are tightly pressed against the steel specimen through a mechanical spring-compression device, and water is used as a coupling medium between the wedge and the steel plate. The scanning is controlled by a robotic arm with a step accuracy of 0.5 mm. The original imaging result of the scanning is shown in Figure 7.

**Figure 8:**
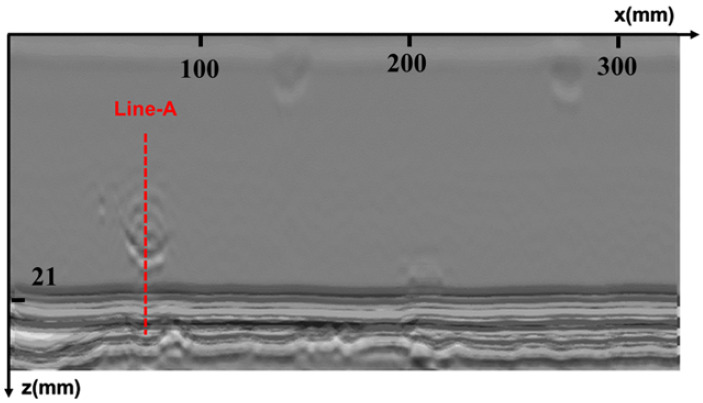
Reconstruction results of time-domain synthetic aperture.

**Figure 9:**
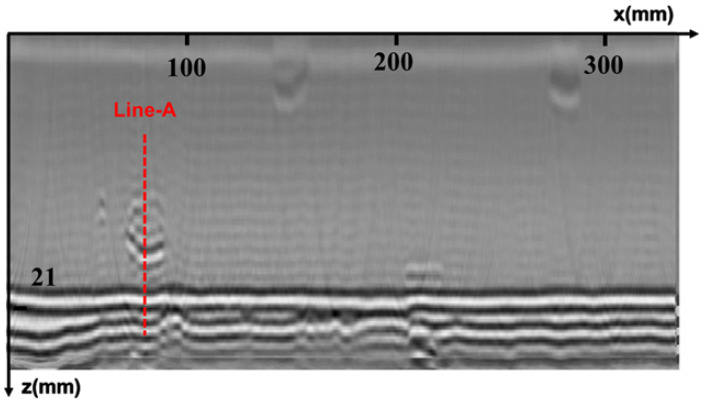
Reconstruction results of frequency-domain synthetic aperture.

**Figure 10:**
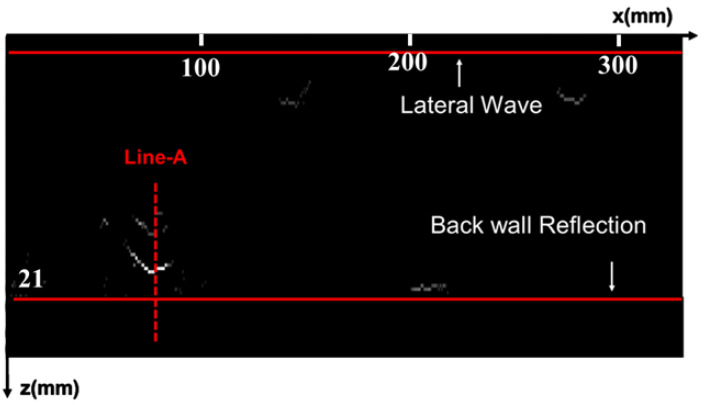
Reconstruction results of frequency-domain synthetic aperture fused with sparse inverse convolution.

**Figure 11:**
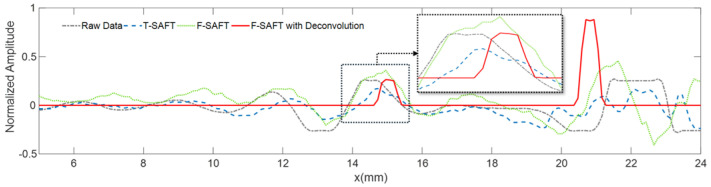
The waveform at Line-A.

**Figure 12:**
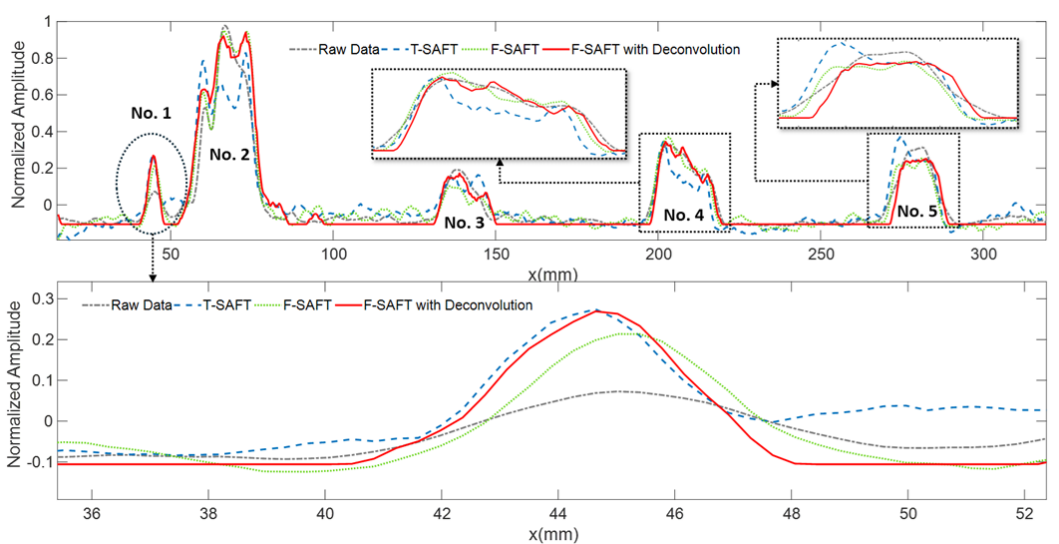
Resolution analysis of imaging results. Upper: Maximum intensity projection of defects. Lower: Zoomed-in image of a slagging defect.

The results of time-domain and frequency-domain synthetic aperture focusing imaging are shown in Figure 8 and Figure 9, respectively, which show that, in the unwelded portion of the root, the frequency-domain imaging method has higher imaging contrast and can separate the defects from the bottom echo. At the same time, the combination of sparse deconvolution and frequency-domain synthesized aperture can further improve the longitudinal resolution, and, as shown in Figure 10, except for the transverse defects that are difficult to identify, the longitudinal resolution of the other four defects is greatly improved, allowing the depths of defects to be clearly located, as shown in Figure 11. In Figure 12, the maximum intensity projection of each defect shows the improvements in the transverse resolutions. Here, the full width at half maximum (FWHM) is used to measure the sizes of defects. The results are listed in Table 1. It can be seen that the mean error of the raw data image for defect length estimation is about 2.2 mm. However, T-SAFT and F-SAFT reduce the errors to 0.9 mm and 0.8 mm, respectively. The reason is that the synthetic aperture process improves resolution by eliminating the ultrasonic diffraction effect. The method of F-SAFT with deconvolution further eliminates the deviation caused by wave vibrations, reducing the error to 0.4 mm. The first defect is relatively small, close to half the size of the probe, and as a result, it can be approximately regarded as a point-like defect for evaluating resolution. The FWHM values of defect 1 from the raw data, T-SAFT, F-SAFT, and F-SAFT with deconvolution are 7 mm, 5 mm, 5 mm, and 4.5 mm, respectively. The proposed method improves the transverse resolution by approximately 35% compared with the raw data. Moreover, the noise level of the image reconstructed by the proposed method is nearly zero. Thanks to the reduced computational volume of the frequency-domain synthetic aperture technique, for the 300 × 1000-point TOFD signal data, the synthetic aperture reconstruction process takes about 0.1 s. Even if the frequency-domain synthetic aperture and sparse inverse convolution fusion reconstruction take about 0.96 s, the reconstruction time remains shorter than the sweeping data acquisition time, meeting the needs of online imaging inspection applications.

### 6.3. Experimental Studies

**Figure 13:**
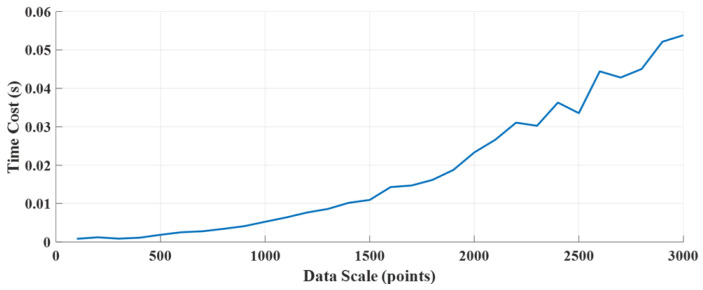
Deconvolution time versus data scale.

**Figure 14:**
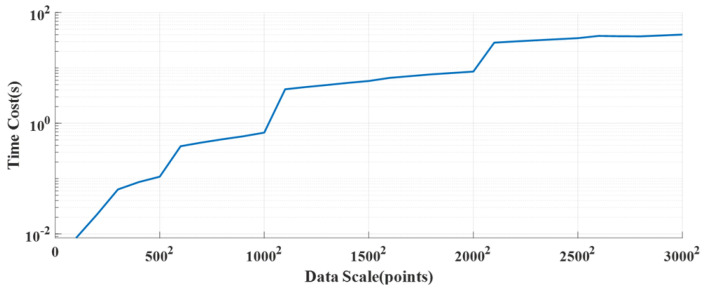
F-SAFT time versus data scale.

During the calculation process, the computational time of the deconvolution part is approximately quadratic with the number of data points. The main reason for this is that matrix multiplication is employed when solving optimization problems using OMP. Therefore, the overall computational complexity is $N^{2}$, as shown in Figure 13. For TOFD imaging, the number of data points usually ranges from 500 to 2000, and the computational time is usually below 0.02 s, which is shorter than the time for a single acquisition in TOFD scanning. On the other hand, the F-SAFT process requires processing the entire image, so it is a two-dimensional process. As the number of data points increases, the computational time of F-SAFT rises in a stepwise manner, as shown in Figure 14. The step points mainly occur at 256, 512, 1024, and 2048. This occurs due to the application of the fast Fourier transform (FFT). When the number of data points is less than a power of 2, zero-padding is applied. In most scanning scenarios, the number of points is less than $1000^{2}$, so the overall computational time is less than 1 s. This is much less than the entire scanning process, which takes about 3~5 min.

## 7. Conclusions

In this paper, an ultrasonic TOFD imaging enhancement technique for pipeline girth welds is proposed. This technique uses reflection coefficients to form images of the defects. The reflection coefficients are obtained by eliminating the electrical and spatial pulse responses with sparse deconvolution and frequency-domain synthetic aperture focusing, respectively. Benefiting from the fast Fourier transform, the enhancement process in the frequency domain significantly reduces computational time. The simulations and experiments demonstrate that using a reflection coefficient to represent the defects in images significantly improves both the longitudinal and transverse resolutions, which enhances the accuracy of defect localization. As a result, the proposed method forms images of the defects with high accuracy and efficiency. However, it does not account for the effects of corrosion, dirt, or irregular pipe surfaces, which can severely degrade images. These factors will be considered further in subsequent theoretical studies. In scenarios such as thick-walled welds, multiple sets of TOFD probes are required for imaging, which increases the demand for parallelism in the imaging algorithm. Therefore, optimization in this regard is also necessary.

## Figures and Tables

**Table 1:** The size estimations of defects.

Defect	True	Raw Data	T-SAFT	F-SAFT	F-SAFT with Deconv.
No. 1	4 mm	7 mm	5 mm	5 mm	4.5 mm
No. 2	20 mm	17 mm	18 mm	19 mm	19 mm
No. 3	17 mm	16 mm	17 mm	16.5 mm	17 mm
No. 4	19 mm	21.5 mm	20 mm	19.5 mm	19.5 mm
No. 5	16 mm	17.5 mm	16.5 mm	16 mm	16 mm
Mean Error	0 mm	2.2 mm	0.9 mm	0.8 mm	0.4 mm

## Data Availability

Data are contained within the article.
